# Supplementary material for: Normalized Neural Representations of Complex Odors
Source: PLoS One. 2016 Nov 11;11(11):e0166456. doi: 10.1371/journal.pone.0166456 (PMC5106022; doi:10.1371/journal.pone.0166456)
Supplement: S1 Appendix — (PDF) [file pone.0166456.s001.pdf]

Supporting Information: Normalized neural representations of complex odors

David Zwicker[1, 2, *]

[1] *School of Engineering and Applied Sciences, Harvard University, Cambridge, MA 02138, USA*
[2] *Kavli Institute for Bionano Science and Technology, Harvard University, Cambridge, MA 02138, USA*

CONTENTS

A. Numerical simulations 1

B. Statistics of normalized concentrations and excitations 1

C. Approximate channel activity 2

D. Odor discriminability 3

E. Receptor binding model 3

A. NUMERICAL SIMULATIONS

We numerically calculated ensemble averages over odors $\boldsymbol{c}$ and sensitivity matrices S_{ni}. Here, we first choose S_{ni} by drawing all entries independently from a log-normal distribution with mean $\bar{S} = 1$ and variance $\mathrm{var}(S_{ni}) = e^{\lambda^2} - 1$. We then draw an odor $\boldsymbol{c}$ using the following procedure: First, we determine which of the N_L ligands are present according to their probabilities p_i. Second, we draw the concentrations c_i for each ligand i that is present from a log-normal distribution with mean μ_i and standard deviation σ_i. We then use Eqs. **1**–**3** given in the main text to map the odor $\boldsymbol{c}$ to a binary activity vector $\boldsymbol{a}$, from which we can for instance calculate the number of active channels. We obtain ensemble averages of such quantities by repeating these steps 10^5 times. This allows us to calculate the mean activities $\langle a_n \rangle$, the covariances $\mathrm{cov}(a_n, a_m)$, and the Pearson correlation coefficient ρ, which is defined as

$$\rho = \frac{1}{{N_\mathrm{R}}^2 - N_\mathrm{R}} \sum_{n \neq m} \frac{\mathrm{cov}(a_n, a_m)}{\left[\mathrm{var}(a_n)\,\mathrm{var}(a_m)\right]^{\frac{1}{2}}} \; . \tag{A.1}$$

We also estimate $P(\boldsymbol{a})$ from an ensemble average to calculate the information I from its definition given in Eq. **4** in the main text.

The resulting statistics only weakly depend on the dimensions N_L and N_R of the input and the output space, respectively, see Fig. A. This is because we consider uncorrelated odors and uncorrelated sensitivities. Conversely, Fig. B shows that these quantities significantly depend on the width λ of the sensitivity distribution, but we only consider the experimentally motivated value $\lambda = 1$ in the main text.

* dzwicker@seas.harvard.edu; http://www.david-zwicker.de

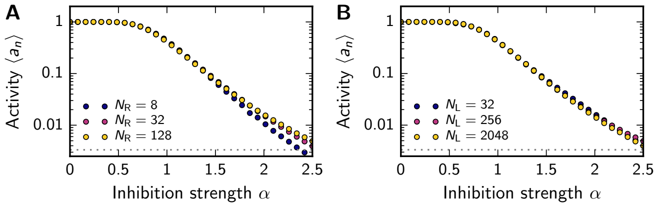

FIG. A. The mean receptor activity $\langle a_n \rangle$ depends only weakly on the receptor count N_R and the number N_L of possible ligands. (A) $\langle a_n \rangle$ as function of the inhibition strengths α for several N_R at $N_\mathrm{L} = 256$. (B) $\langle a_n \rangle$ as function of α for several N_L at $N_\mathrm{R} = 32$. (A–B) Remaining parameters are $p_i = 25.6\,{N_\mathrm{L}}^{-1}$, $\mu_i = \sigma_i = 1$, and $\lambda = 1$.

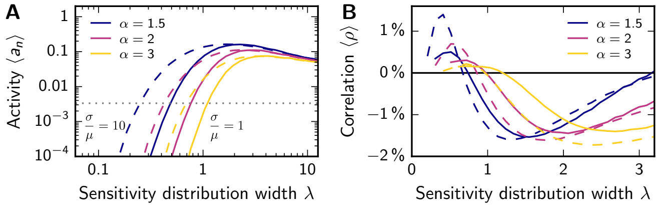

FIG. B. Influence of the width λ of the sensitivity distribution on the statistics of the odor representations. (A) Expected channel activity $\langle a_n \rangle$ as a function of λ for several inhibition strengths α. Intermediated values, $\lambda \approx 1$, lead to larger activities. (B) Mean Pearson correlation coefficient $\langle \rho \rangle$ calculated from an ensemble average of Eq. A.1 as a function of λ for several α. For small λ, $\langle a_n \rangle$ was too small to estimate ρ reliably. (A–B) Results are shown for small ($\frac{\sigma}{\mu} = 1$, solid lines) and large ($\frac{\sigma}{\mu} = 10$, dashed lines) concentration variability. Remaining parameters are $N_\mathrm{R} = 32$, $N_\mathrm{L} = 256$, and $p_i = 0.1$.

B. STATISTICS OF NORMALIZED CONCENTRATIONS AND EXCITATIONS

Let p_i be the probability that ligand i is present in an odor. If it is present, its concentration c_i is drawn from a log-normal distribution with mean μ_i and standard deviation σ_i, while $c_i = 0$ if the ligand is not present. Hence,

$$\langle c_i \rangle = p_i \mu_i \tag{B.2a}$$

$$\mathrm{var}(c_i) = (p_i - p_i^2)\mu_i^2 + p_i \sigma_i^2 \; , \tag{B.2b}$$

while the covariances $\mathrm{cov}(c_i, c_j) = \langle c_i c_j \rangle - \langle c_i \rangle \langle c_j \rangle$ vanish for $i \neq j$ since the ligands are independent. The statistics

of the total concentration $c_{\rm tot} = \sum_i c_i$ read

$$\langle c_{\rm tot}\rangle = \sum_{i=1}^{N_{\rm L}} \langle c_i\rangle \quad \text{and} \quad \mathrm{var}(c_{\rm tot}) = \sum_{i=1}^{N_{\rm L}} \mathrm{var}(c_i)\ . \tag{B.3}$$

The excitations e_n are given by $e_n = \sum_i S_{ni} c_i$, where the sensitivities S_{ni} are log-normally distributed with mean $\langle S_{ni}\rangle = \bar{S}$ and variance $\mathrm{var}(S_{ni}) = \bar{S}^2(e^{\lambda^2} - 1)$. Hence,

$$\langle e_n\rangle = \bar{S}\langle c_{\rm tot}\rangle \tag{B.4a}$$

$$\mathrm{var}(e_n) = \bar{S}^2\,\mathrm{var}(c_{\rm tot}) + \mathrm{var}(S_{ni}) \sum_{i=1}^{N_{\rm L}} \langle c_i^2\rangle\ , \tag{B.4b}$$

where $\langle c_i^2\rangle = p_i(\mu_i^2 + \sigma_i^2)$ and $\mathrm{cov}(e_n, e_m) = 0$ for $n \neq m$.

We next determine the statistics of the normalized concentrations $\hat{c}_i = c_i/c_{\rm tot}$. For simplicity, we consider large odors, $\sum_i p_i \gg 1$, where $c_{\rm tot}$ can be considered as an independent random variable. Since $c_{\rm tot}$ is the sum of (a variable) number of log-normally distributed random variables, its distribution can be approximated by another log-normal distribution [2], which we parameterize by its mean $\mu_{\rm tot}$ and variance $\sigma_{\rm tot}^2$. We consider the simple approximation where these parameters are directly given by Eq. B.3 [1]. This choice approximates the tail of the distribution well, but leads to errors in the vicinity of the mean [2].

Since both $c_{\rm tot}$ and c_i are log-normally distributed when ligand i is present in an odor ($c_i > 0$), $\hat{c}_i$ is also log-normally distributed in this case and

$$\langle \hat{c}_i\rangle_{c_i>0} = \frac{\mu_i}{\mu_{\rm tot}}\chi \tag{B.5a}$$

$$\mathrm{var}(\hat{c}_i)_{c_i>0} = \frac{\mu_i^2\chi^2}{\mu_{\rm tot}^2}\left(\frac{\sigma_i^2}{\mu_i^2}\chi + \chi - 1\right)\ , \tag{B.5b}$$

where $\chi = 1 + \sigma_{\rm tot}^2\mu_{\rm tot}^{-2}$. Since $\hat{c}_i = 0$ with probability $1 - p_i$, the statistics of $\hat{c}_i$ read

$$\langle \hat{c}_i\rangle = \frac{p_i\mu_i}{\mu_{\rm tot}}\chi \tag{B.6a}$$

$$\mathrm{var}(\hat{c}_i) = \frac{p_i\mu_i^2\chi^2}{\mu_{\rm tot}^2}\left(\frac{\sigma_i^2}{\mu_i^2}\chi + \chi - p_i\right)\ . \tag{B.6b}$$

Note that the covariance $\mathrm{cov}(\hat{c}_i, \hat{c}_j)$ does not vanish since the $\hat{c}_i$ are not independent. In particular, $\mathrm{var}(\sum_i \hat{c}_i) = 0$, since $\sum_i \hat{c}_i = 1$ by definition. This condition is only consistent with Eq. B.6a if $\chi \approx 1$, which implies that $c_{\rm tot}$ must not vary much, $\frac{\sigma_{\rm tot}}{\mu_{\rm tot}} \ll 1$. Using $\chi = 1$, the statistics of the normalized excitations $\hat{e}_n = \bar{S}^{-1}\sum_i S_{ni}\hat{c}_i$ read

$$\langle \hat{e}_n\rangle = 1 \tag{B.7a}$$

$$\mathrm{var}(\hat{e}_n) = \frac{\mathrm{var}(S_{ni})}{\bar{S}^2}\left\langle \sum_i \hat{c}_i^2 \right\rangle\ , \tag{B.7b}$$

where $\langle \sum_i \hat{c}_i^2\rangle \approx \sum_i \langle \hat{c}_i^2\rangle$ with $\langle \hat{c}_i^2\rangle = \langle \hat{c}_i\rangle^2 + \mathrm{var}(\hat{c}_i)$ and the statistics given in Eq. B.6.

In the simple case where all ligands are drawn from the same distribution ($p_i = p$, $\mu_i = \mu$, $\sigma_i = \sigma$), we obtain

$$\langle \hat{c}_i\rangle \approx \frac{1}{N_{\rm L}}\ , \qquad \mathrm{var}(\hat{c}_i) \approx \frac{1 - p + \frac{\sigma^2}{\mu^2}}{sN_{\rm L}}\ , \tag{B.8}$$

and $\langle \hat{c}_i^2\rangle \approx \frac{1}{sN_{\rm L}}(\frac{\sigma^2}{\mu^2} + 1)$, such that

$$\mathrm{var}(\hat{e}_n) \approx \frac{1}{s}\left(1 + \frac{\sigma^2}{\mu^2}\right)\frac{\mathrm{var}(S_{ni})}{\bar{S}^2}\ , \tag{B.9}$$

which is equivalent to Eq. **5** in the main text.

C. APPROXIMATE CHANNEL ACTIVITY

We estimate the expected activity $\langle a_n\rangle$ by the probability that the normalized excitations $\hat{e}_n$ exceed the expected normalized threshold α. Since both the sensitivities S_{ni} and the normalized concentrations $\hat{c}_i$ are approximately log-normally distributed, $\hat{e}_n$ can also be approximated by a log-normal distribution [1]. The associated probability distribution function reads

$$f(\hat{e}_n) = \frac{1}{\sqrt{2\pi}S_n\hat{e}_n}\exp\left[-\frac{\big(M_n - \ln(\hat{e}_n)\big)^2}{2S_n^2}\right] \tag{C.10}$$

and the cumulative distribution function is

$$F(\hat{e}_n) = \frac{1}{2}\,\mathrm{erfc}\left[\frac{M_n - \ln(\hat{e}_n)}{\sqrt{2}S_n}\right]\ . \tag{C.11}$$

The parameters M_n and S_n can be determined from the mean and variance

$$\langle \hat{e}_n\rangle = \exp\left(M_n + \frac{S_n^2}{2}\right) \tag{C.12a}$$

$$\mathrm{var}(\hat{e}_n) = e^{2M_n + S_n^2}\left(e^{S_n^2} - 1\right)\ . \tag{C.12b}$$

Solving these equations for M_n and S_n, we obtain

$$M_n = \ln\langle \hat{e}_n\rangle - \zeta \qquad \text{and} \qquad S_n = \sqrt{2\zeta}\ , \tag{C.13}$$

where $\zeta = \frac{1}{2}\ln(1 + \mathrm{var}(\hat{e}_n)\langle \hat{e}_n\rangle^{-2})$. Eq. **6** of the main text follows from this and Eq. **5**. For small $\langle a_n\rangle$ we have

$$\langle a_n\rangle \approx \frac{2\sqrt{\zeta/\pi}}{\ln(\alpha) + \zeta}\exp\left[-\frac{(\ln(\alpha) + \zeta)^2}{4\zeta}\right]\ , \tag{C.14}$$

which follows from $\mathrm{erfc}(x) \approx e^{-x^2}/(x\sqrt{\pi})$, valid for $x \gg 1$. For small ζ, we obtain the approximate scaling $\ln\langle a_n\rangle \sim -(\ln\alpha)^2/(4\zeta)$, where $\zeta \sim s^{-1}$ for $s \gg 1$.

Fig. 2C of the main text shows that Eq. C.14 approximates the channel activity $\langle a_n\rangle$ very well. Moreover, this approximation together with Eq. (7) of the main text can be used to estimate the transmitted information I. Fig. CA shows that this also approximates the true numerical values very well.

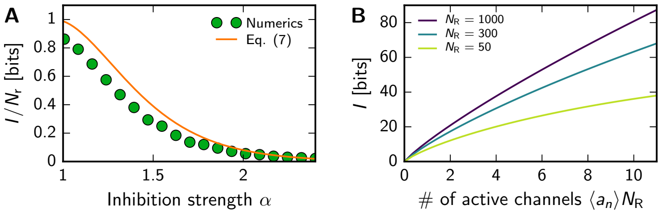

FIG. C. Stronger inhibition reduces the transmitted information I. (A) I as function of the inhibition strength α from numerical ensemble averages of Eq. (4) of the main text (green dots) and the approximation from Eq. C.14 together with Eq. (7) of the main text (solid orange line). Model parameters are $N_\mathrm{R} = 16$, $N_\mathrm{L} = 128$, $p_i = 0.25$, $\mu_i = \sigma_i = 1$, and $\lambda = 1$. (B) I calculated using Eq. (7) of the main text as a function of the number $\langle a_n \rangle N_\mathrm{R}$ of active channels for several N_R.

D. ODOR DISCRIMINABILITY

We quantify the discriminability of two odors by the Hamming distance d of their respective representations $\boldsymbol{a}$ for several different cases:

a. Uncorrelated odors The expected distance $\langle d \rangle$ between the activity patterns $\boldsymbol{a}^{(1)}$ and $\boldsymbol{a}^{(2)}$ of two independent odors is

$$\langle d \rangle = N_\mathrm{R} \left(\langle a_n^{(1)} \rangle + \langle a_n^{(2)} \rangle - 2 \langle a_n^{(1)} \rangle \langle a_n^{(2)} \rangle \right) , \qquad \text{(D.15)}$$

where $\langle a_n^{(1)} \rangle$ and $\langle a_n^{(2)} \rangle$ denote the expected activities of the two odors, averaged over sensitivity matrices, and we neglect correlations $\mathrm{cov}(a_n, a_m)$ for simplicity.

b. Adding target to background We calculate the expected change $\langle d \rangle$ of the representation when a target odor $\boldsymbol{c}^\mathrm{t}$ is added to a background odor $\boldsymbol{c}^\mathrm{b}$. Because the odor concentrations are specified, we consider the actual excitations e_n instead of the normalized quantities $\hat{e}_n$. Taking an ensemble average over sensitivity matrices, the excitations associated with the two odors are characterized by probability distribution functions $f_E^\mathrm{t}(e^\mathrm{t})$ and $f_E^\mathrm{b}(e^\mathrm{b})$ for the target and the background, respectively. We here consider log-normally distributed e_n, which are parameterized by their mean and variance,

$$\langle e_n \rangle = \bar{S} \sum_{i=1}^{N_\mathrm{L}} c_i \qquad \mathrm{var}(e_n) = \mathrm{var}(S_{ni}) \sum_{i=1}^{N_\mathrm{L}} c_i^2 , \qquad \text{(D.16)}$$

where $\mathrm{var}(S_{ni}) = \bar{S}^2 (e^{\lambda^2} - 1)$.

When the target is added to the background, the expected threshold $\langle \gamma \rangle$ increases from $\gamma^\mathrm{b} = \alpha \langle e^\mathrm{b} \rangle$ to $\gamma^\mathrm{s} = \alpha(\langle e^\mathrm{b} \rangle + \langle e^\mathrm{t} \rangle)$, where $\langle e^\kappa \rangle$ denotes the mean excitation $\langle e^\kappa \rangle = \int z \, f_E^\kappa(z) \, \mathrm{d}z$ for $\kappa = \mathrm{t}, \mathrm{b}$. This increase in the threshold can deactivate a channel if it was previously active, i.e. if its excitation was larger than the threshold associated with the background, $e^\mathrm{b} > \gamma^\mathrm{b}$. For such e^b, the probability that the receptor gets deactivated by adding the target is $P(e^\mathrm{b} + e^\mathrm{t} < \gamma^\mathrm{s} | e^\mathrm{b})$. Integrating over all possible e^b, we thus get the probability p_off that a channel becomes inactive,

$$\begin{aligned} p_\mathrm{off} &= \int_{\gamma^\mathrm{b}}^\infty P\left(e^\mathrm{b} + e^\mathrm{t} < \gamma^\mathrm{s} \,\middle|\, e^\mathrm{b}\right) f_E^\mathrm{b}\left(e^\mathrm{b}\right) \mathrm{d}e^\mathrm{b} \\ &= \int_{\gamma^\mathrm{b}}^\infty F_E^\mathrm{t}\left(\gamma^\mathrm{s} - e^\mathrm{b}\right) f_E^\mathrm{b}\left(e^\mathrm{b}\right) \mathrm{d}e^\mathrm{b} , \end{aligned} \qquad \text{(D.17)}$$

where $F_E^\mathrm{t}(e^\mathrm{t})$ is the cumulative distribution function associated with $f_E^\mathrm{t}(e^\mathrm{t})$. Conversely, a channel becomes active when the additional excitation by the target odor brings it above the threshold γ^s. The associated probability p_on reads

$$p_\mathrm{on} = \int_0^{\gamma^\mathrm{b}} \left[1 - F_E^\mathrm{t}\left(\gamma^\mathrm{s} - e^\mathrm{b}\right)\right] f_E^\mathrm{b}\left(e^\mathrm{b}\right) \mathrm{d}e^\mathrm{b} . \qquad \text{(D.18)}$$

Taken together, the expected number $\langle d \rangle$ of channels that change their state reads

$$\langle d \rangle = N_\mathrm{R} \cdot \left(p_\mathrm{on} + p_\mathrm{off}\right) . \qquad \text{(D.19)}$$

There are three simple limits that we can solve analytically: If there is no target, $\langle e^\mathrm{t} \rangle = 0$, the activation pattern does not change and we have $\langle d \rangle = 0$. In the opposing limit of a dominant target, $\langle e^\mathrm{t} \rangle \to \infty$, the activation patterns are independent and we recover the distance $\langle d \rangle_\mathrm{max}$ for uncorrelated odors, which is given by Eq. D.15. Lastly, in the case where the target and the background are identically distributed, $\langle e^\mathrm{b} \rangle = \langle e^\mathrm{t} \rangle$ and $\mathrm{var}(e^\mathrm{b}) = \mathrm{var}(e^\mathrm{t})$, we have $\langle d \rangle = \frac{1}{2} \langle d \rangle_\mathrm{max}$.

c. Discriminating two odors of equal size We consider the simple case of two odors that each contain s ligands at equal concentration, sharing s_b of them, such that the expected threshold $\langle \gamma \rangle$ is the same for both odors. Similar to the derivation above, we here calculate the probability p that a channel is active for one odor, but not for the other. The s_b ligands that are present in both odors cause a baseline excitation e^b, which is distributed according to $f_E^\mathrm{b}(e^\mathrm{b})$. A channel is inactive for an odor with probability $F_E^\mathrm{d}(\langle \gamma \rangle - e^\mathrm{b})$, where $F_E^\mathrm{d}(e^\mathrm{d})$ is the cumulative distribution function of the excitation caused by the $s_\mathrm{d} = s - s_\mathrm{b}$ different ligands. Hence,

$$p = 2 \int_0^{\langle \gamma \rangle} F_E^\mathrm{d}(z) \left[1 - F_E^\mathrm{d}(z)\right] f_E^\mathrm{b}(e^\mathrm{b}) \mathrm{d}e^\mathrm{b} , \qquad \text{(D.20)}$$

where $z = \langle \gamma \rangle - e^\mathrm{b}$. Note that the upper bound of the integral is $\langle \gamma \rangle$ since channels will be active for both odors if $e^\mathrm{b} \geq \langle \gamma \rangle$. The associated Hamming distance $\langle d \rangle$ between the two odors is then given by $\langle d \rangle = p N_\mathrm{R}$.

E. RECEPTOR BINDING MODEL

We consider a simple model where receptors R_n get activated when they bind ligands L_i. This binding

is described by the chemical reaction $R_n + L_i \rightleftharpoons R_nL_i$, where R_nL_i is the receptor-ligand complex. In equilibrium, the concentrations denoted by square brackets obey $[R_nL_i] = K_{ni}\cdot[R_n][L_i]$, where K_{ni} is the binding constant of the reaction. Hence,

$$[R_nL_i] = \frac{c_n^{\rm rec} K_{ni} c_i}{1+\sum_i K_{ni} c_i} \; , \tag{E.21}$$

where we consider the case where multiple ligands compete for the same receptor. Here, $c_i = [L_i]$ is the concentration of free ligands and $c_n^{\rm rec} = [R_n] + \sum_i [R_nL_i]$ denotes the fixed concentration of receptors, which is related to the copy number of receptors of type n. We consider a simple receptor model where the excitation is proportional to the concentration of the bound ligands, such that the excitation accumulated in glomerulus n reads

$$e_n = \beta_n \frac{N_n^{\rm rec}}{c_n^{\rm rec}} \sum_{i=1}^{N_{\rm L}} [R_nL_i] = \beta_n N_n^{\rm rec} \frac{\sum_i K_{ni} c_i}{1+\sum_i K_{ni} c_i} \; . \tag{E.22}$$

Here, $N_n^{\rm rec}$ is the copy number of receptors of type n and β_n characterizes their excitability, which could for instance be modified by point mutations [3]. Defining $S_{ni} = \beta_n N_n^{\rm rec} K_{ni}$, we recover Eq. **1** of the main text in the limit of small concentrations, $\sum_i K_{ni} c_i \ll 1$. The sensitivities are thus proportional to the copy number $N_n^{\rm rec}$ and the biochemical details encoded in $\beta_n K_{ni}$.

[1] Fenton, L. F., Communications Systems, IRE Transactions on **8**, 57 (1960).

[2] Wu, J., Mehta, N. B., and Zhang, J., in *GLOBECOM '05. IEEE Global Telecommunications Conference, 2005.*, Vol. 6 (2005) pp. 3413–3417.

[3] Yu, Y., Claire, A., Ni, M. J., Adipietro, K. A., Golebiowski, J., Matsunami, H., and Ma, M., Proc. Natl. Acad. Sci. USA **112**, 14966 (2015).
